# Differential gene expression, including *Sjfs800*, in *Schistosoma japonicum* females at pre-pairing, initial pairing and oviposition

**DOI:** 10.1186/s13071-019-3672-8

**Published:** 2019-08-23

**Authors:** Fengchun Liu, Han Ding, Jiaming Tian, Congyu Zhou, Fei Yang, Wei Shao, Yinan Du, Xin Hou, Cuiping Ren, Jijia Shen, Miao Liu

**Affiliations:** 1 Department of Microbiology and Parasitology, School of Basic Medical Sciences, Anhui Medical University, 81# Meishan Road, Hefei, 230032 Anhui People’s Republic of China; 2 Anhui Provincial Laboratory of Microbiology and Parasitology, Anhui Medical University, 81# Meishan Road, Hefei, 230032 Anhui People’s Republic of China

**Keywords:** *Schistosoma japonicum*, *Sjfs800*, Vitellarium development, Egg production

## Abstract

**Background:**

Schistosomiasis is a prevalent but neglected tropical disease caused by parasitic trematodes of the genus *Schistosoma*, with the primary disease-causing species being *S. haematobium*, *S. mansoni* and *S. japonicum*. Male–female pairing of schistosomes is necessary for sexual maturity and the production of a large number of eggs, which are primarily responsible for schistosomiasis dissemination and pathology.

**Methods:**

Here, we used microarray hybridization, bioinformatics, quantitative PCR, *in situ* hybridization and gene silencing assays to identify genes that play critical roles in *S. japonicum* reproduction biology, particularly in vitellarium development, a process that affects male–female pairing, sexual maturation and subsequent egg production.

**Results:**

Microarray hybridization analyses generated a comprehensive set of genes differentially transcribed before and after male–female pairing. Although the transcript profiles of females were similar 16 and 18 days after host infection, marked gene expression changes were observed at 24 days. The 30 most abundantly transcribed genes on day 24 included those associated with vitellarium development. Among these, the gene for female-specific 800 (*fs800*) was substantially upregulated. Our *in situ* hybridization results in female *S. japonicum* indicated that *Sjfs800* mRNA was observed only in the vitellarium, localized in mature vitelline cells. Knocking down the *Sjfs800* gene in female *S. japonicum* by approximately 60% reduced the number of mature vitelline cells, decreased rates of pairing and oviposition, and decreased the number of eggs produced in each male–female pairing by about 50%.

**Conclusions:**

These results indicate that *Sj*fs800 may play a role in vitellarium development and egg production in *S. japonicum* and suggest that *Sj*fs800 regulation may provide a novel approach for the prevention or treatment of schistosomiasis.

## Background

Schistosomiasis is a tropical disease caused by parasitic trematodes of the genus *Schistosoma*. Although it is one of the most prevalent tropical infectious diseases, with more than 240 million people in 78 countries infected and approximately 800 million people at risk, schistosomiasis has been drastically understudied [1–3]. The primary disease-causing species of *Schistosoma* are *S. haematobium*, *S. mansoni* and *S. japonicum*, the latter of which is distributed in China, Indonesia and the Philippines [1–3]. Disease burden assessments for schistosomiasis, based on the extent of end-organ damage and the associated morbidities related to malnutrition and chronic inflammation, indicate that the annual number of disability-adjusted life years lost is approximately 70 million [4]. Current control of schistosomiasis depends largely on a single drug, praziquantel; however, reliance on a single drug produces a precarious situation. Indeed, some studies have shown that isolates of schistosomes have reduced susceptibility to praziquantel [5–7]. Thus, additional novel strategies are urgently needed to prevent and control schistosomiasis.

*Schistosoma japonicum* has a complex developmental cycle that involves an aquatic snail as an intermediate host and a mammalian definitive host. In contrast to other trematode species, these parasites are unique in that males and females need to pair to continue development. Pairing of schistosome females and males promotes female reproductive system maturation and the production of eggs, which are a primary means of schistosomiasis transmission and immunopathological lesions [8–10]. Maturation and maintenance of normal reproductive function in female schistosome require permanent pairing with the male. During pairing, germ cells in the reproductive organ differentiate into oocytes or vitellocytes, and some chemical or tactile stimulus exchange occurs between the male and female, leading to a cascade of changes during the pairing process [11–16]. However, the effects on female reproductive system development and the molecular mechanisms underpinning male–female pairing have not been completely determined, leaving myriad questions that require further study.

Ongoing work in our laboratory has indicated that during the development of *S. japonicum*, no male–female pairing occurs up to 16 days after the host is infected. Some pairing occurs 17 days post-infection (dpi), and pairing is common 18 dpi. Paired females begin laying eggs approximately 24 dpi [17, 18]. Therefore, in the present study, to identify genes that likely contribute to pairing and reproduction, we used microarray technology to determine differential gene expression in females 16, 18 and 24 dpi. We identified genes that play critical roles in the development of the vitellarium and in the production of eggs, providing a clearer understanding of gene regulation before and after male–female pairing in the *S. japonicum* female and insights on schistosome reproduction biology.

## Methods

### Animals and parasites

Freshly shed wild-type cercariae of *S. japonicum* were harvested from infected *Oncomelania hupensis* that were purchased from the Hunan Institute of Parasitic Diseases in Yueyang, China. Female Kunming mice (6–8 weeks-old) and New Zealand rabbits (4 months-old) were obtained from the Laboratory Animal Center of Anhui Medical University. New Zealand rabbits and female Kunming mice were infected with 1000 or 50 cercariae, respectively, *via* the skin of the abdomen. After 16, 18, 24, 28 or 42 dpi the worms were perfused from the hepatic portal vein using perfusion techniques. Male and female worms were manually separated. In order to collect *S. japonicum* eggs, liver tissues from rabbits 6 weeks post-infection were homogenized and then subjected to consecutive fractional filtration. The filtrate was centrifuged. The supernatant and the tissue-containing layers were removed, leaving the egg-containing layer, which was diluted in 1.2% saline and passed through a nylon net (300 mesh, i.e., 300 holes per inch). All parasite samples were soaked in RNAlater (Invitrogen, Carlsbad, CA, USA) and stored at − 80 °C until they were used for total RNA extraction.

### RNA extraction, amplification and labeling

Total RNA was extracted and purified using RNeasy Micro Kit (Qiagen, Hilden, Germany) following the manufacturer’s instructions, and the overall RNA quality was assessed using denaturing gel electrophoresis (Agilent Technologies, Santa Clara, CA, USA). Total RNA was amplified and labeled using a Low Input Quick Amp Labeling Kit, one-color (Agilent Technologies), following the manufacturer’s instructions. Labeled cRNA was purified using an RNeasy Mini Kit (Qiagen).

### Microarray construction and hybridization and subsequent data analysis

A schistosome genome-wide microarray was used for profiling gene expression in *S. japonicum* females 16, 18 and 24 dpi. Microarrays were printed on the Agilent custom *Schistosoma* 4 × 44K chip (design ID: 048766). The chip sequence is shown in Additional file 1: Table S1. There was a total of eight specimens. There were three specimens of 16 dpi female worms, three specimens of 18 dpi female worms and two specimens of 24 dpi female worms. Thus, two chips in total were needed. Each slide was hybridized with 1.65 μg of Cy3-labeled cRNA using a Gene Expression Hybridization Kit (Agilent Technologies) with a Hybridization Oven (Agilent Technologies) according to the manufacturer’s instructions. After 17 h of hybridization had elapsed, the slides were washed in staining dishes (Thermo Shandon, Waltham, MA, USA) with a Gene Expression Wash Buffer Kit (Agilent Technologies), following the manufacturers’ instructions. All of these aforementioned procedures were performed by SHBIO Biotechnology Corporation (Shanghai, China). The slides were scanned with an Agilent Microarray Scanner (Agilent Technologies) using the following default settings: dye channel, green; scan resolution, 5 μm; photomultiplier tube, 100% and 10%, 16 bit. Data were extracted with Feature Extraction software v. 10.7 (Agilent Technologies). Raw data were normalized using the Quantile algorithm, GeneSpring software v.11.0 (Agilent Technologies). Outlier probes were identified, and their contribution was reduced at the reported gene expression level. The expression value of a gene was a weighted average of all forward or reverse probe sets when both background correction and quantile normalization were performed.

### Bioinformatics analysis

The mRNA and expressed sequence tag transcripts highly enriched in *S. japonicum* 16, 18, and 24 dpi were retrieved from the National Center for Biotechnology Information Entrez Gene database (http://www.ncbi.nlm.nih.gov/gene) based on fold change (FC = signal A/signal B) values (FC ≥ 2, three biological replicates; FC ≥ 3, two biological replicates). Student’s t-test was used to determine genes differentially expressed between one stage and the other. To determine the potential function of these upregulated genes, the Gene Ontology (GO) functional categories were assessed. All analyses were conducted using the online SBC Analysis System of SHBIO Biotechnology Corporation (http://sas.shbio.com). The raw data were normalized using the quantile algorithm from the limma package in R. Heatmaps representing differentially regulated genes were generated using Cluster v.3.0 and Tree View software. Two sets of samples were generated using DiffGenes differential expression analysis package in R. The first set of samples was in one column, and the second set of samples was in a second column. The *P-*value was set to the desired range of statistical significance, and the fold-change was set to the desired difference. The gene functions of those genes identified with the desired fold change and *P-*values were then determined and annotated.

### Quantitative PCR (qPCR)

Thirteen genes whose expression levels were increased and two genes whose expression levels were decreased in females’ initial pairing and oviposition (i.e. 18 and 24 dpi) relative to those levels pre-pairing (i.e. 16 dpi) were selected for validation using qPCR. Total RNA (500 ng) from the females was reverse transcribed into first-strand cDNA using a PrimeScript RT Reagent Kit (TaKaRa, Dalian, China) according to the manufacturer’s instructions. Each 20 μl of PCR reaction contained 10 μl of 2× SYBR Premix Ex qTaq II, 1 μl of cDNA, 1.6 μl of the forward and reverse primer pair, 0.4 μl of 50× ROX Reference Dye and 7 μl of sterile water. The PCR cycling conditions were as follows: 95 °C for 30 s, followed by 40 cycles of 30 s denaturation at 95 °C and 1 min annealing and extension at 60 °C. A dissociation step (95 °C for 15 s, 60 °C for 1 min and 95 °C for 15 s) was performed to confirm the amplification specificity for each gene. A reliable reference gene for transcriptomic analysis of *S. japonicum*, proteasome 26S subunit, non-ATPase 4 (*PSMD4*), was used as a control gene in the assays [19]. The PCR primers were designed using Primer Premier 5 software (Table 1). PCR reactions were performed in technical triplicates using the StepOnePlus Real-Time PCR System (Applied Biosystems, Foster City, CA, USA). The relative expression level of each gene was analyzed using SDS v.1.4 software (Applied Biosystems).

**Table 1:** Quantitative PCR primers (5’ to 3’)

Gene name	Gene ID	Sense primer	Anti-sense primer
Unknown	226471383	ATTCACCACAACCCACT	TATTGCCACCTCCACTT
Trematode eggshell synthesis	226473101	ATCCACGTCTTACCAGA	AGCAGCGATACTACCTT
Superoxide dismutase	226474963	CCATTCAACATGCGTCC	CTGCCTTCATCTGGATTTCT
Keratin 9	226477615	ACAGCTACGGAAATGC	CAATAATAGGAGGGTGC
Prostatic spermine-binding protein	226477985	GATTATGCTGATGTGAG	CGTTTGATTCGTGTCTA
Myoglobin 1	226487201	TCAGGGACTTGATGCTA	CAACTGGTCGAGTTCTAT
Annexin	226469397	TCCATTCGGTTTACTCT	TTCAGCAATGTCCCTAG
Cytochrome *b*	56046805	AAGAAGTTGGTGGTGG	GTGCCTTTGAGGTTGTCC
Female-specific protein 800	60600619	TGGAAACGAAAGTGAG	CTGGAATTGAAAGGACC
Tetraspanin-1	56757404	AAGGTAAAGGTGGTAGC	CAATGAATGCCGATAAG
Tyrosinase precursor	56757710	TGGTGTTTGTTTCCCTA	CTCTATTACCACCTCTTTGA
Putative transmembrane protein	56757833	TAGTTGGGAGACTTTGC	TACATTTCGGATTGCTG
Multivesicular body protein 5	226467851	TTGCTTCAAGGCGGCT	CCGTGTTGTTTATTGGGAC
Unknown gene	226477693	TGGTGGTCCAGATTGTT	CATAGTAGTCATTTCCGTAG
Egghell precursor protein	226478597	ATGACTACAACTCCGACTAC	CTCTGACATCTAAACGACCA

Total RNAs from eggs, cercariae, schistosomula and females at 24 and 42 dpi were extracted using TRIzol reagent (Life Technologies, Carlsbad, CA, USA) following the manufacturer’s instructions. Total RNA concentration and purity were measured using a NanoDrop 2000 (Thermo Fisher Scientific, Waltham, MA, USA). Quantitative PCR was performed as described above using primer (Table 1) combinations to amplify gene transcripts of *S. japonicum* female-specific 800 (*fs800*).

### *In situ* hybridization

Riboprobes were synthesized according to previously published methods [20]. Briefly, Transcripts were amplified from mixed adult male and female cDNA using the following primers: sense primer (5′-GAA TTC TGC CCA TAG GAA TGG TAG AAT-3′); anti-sense primer (5′-AAG CTT CCT CAC TGT TGT TAG GCG AA-3′). PCR products were cloned into the pSPT-18/19 T vector. Probes were synthesized from restriction enzyme-digested DNA according to the orientation of the insert in Pspt-18/19 using a DIG RNA Labeling Kit (SP6/T7) (Roche, Mannheim, Germany) labeled with digoxigenin. For whole-mount *in situ* hybridization, trematodes were fixed in 4% paraformaldehyde for 45 min and dehydrated in methanol. Following bleaching in 6% hydrogen peroxide in methanol to prevent tanning of the vitellaria, trematodes were permeabilized using proteinase k (TaKaRa), incubated with prehybridization buffer (50% deionized formamide, 5× saline sodium citrate, 1 mg/ml yeast RNA, 1% Tween 20) for 2 h and then hybridized (pre-hybridization buffer with 10% dextran sulfate) with a riboprobe at 56 °C for 20 h. Excess riboprobe was removed by washing in 2× and 0.2× saline sodium citrate, followed by blocking in blocking reagent (Roche). The bound riboprobe was detected after incubation of trematodes in anti-digoxigenin alkaline phosphatase-conjugated antibody (Roche) diluted 1:5000 in blocking reagent overnight at 4 °C. The unbound antibody was removed by washing in maleic acid buffer (100 mM maleic acid, 150 mM NaCl and 0.1% Tween 20 at pH 7.5) for 4 h in 12 changes of buffer. After washing, specimens were incubated in detection buffer (0.1 M Tris-HCl and 0.1 M NaCl at pH 9.5). Hybridization signals were detected by adding 200 μl of NBT/BCIP in detection buffer. After development, trematodes were washed in PBS then de-stained with 100% ethanol. Trematodes were mounted in 80% glycerol, and then microscopy and digital image capture were performed using an Olympus DP73 microscope (Olympus, Tokyo, Japan). All images were obtained from at least three independent WISH experiments using at least 20 worms of each sex in each experiment.

### RNA interference

The siRNAs (21 base pairs) were designed using the *Sjfs800* mRNA sequence (GenBank accession no. FN313803.1) with the Thermo Fisher website software (https://rnaidesigner.thermofisher.com) and chemically synthesized by Shanghai GenePharma Co., Ltd. (Shanghai, China). Three siRNA sequences (siRNA1-3) that shared no homology with any other *S. japonicum* gene based on an online analysis with BLAST (National Center for Biotechnology Information) and the scrambled siRNA sequence are shown in Table 2. The results of RNAi were tested using qPCR as previously described [21]. Briefly, Kunming mice were challenged percutaneously with 60–70 cercariae and were humanely killed 28 dpi. The worms were obtained by portal perfusion using RPMI 1640 medium at 37 °C and were then incubated in 24-well plates (15 pairs/well) containing 1 ml of complete Basch medium, with half of the medium exchanged every day. The medium was supplemented with 10 KU/ml penicillin, 10 mg/ml streptomycin, 250 μg/ml amphotericin B (Sangon Biotech, Shanghai, China) and 10% fetal bovine serum (Gibco, Grand Island, NY, USA). Lipofectamine RNAiMAX Transfection Reagent (Invitrogen) was used to transfect worms with one of the three *Sjfs800*-specific siRNAs, at a final concentration of 100 nM, or RNAase-free water (mock, no siRNA). The gene-silencing effect of each siRNA was determined using qPCR at the end of a 72-h cultivation period. The worms were then transfected with either the siRNA that was found to be most efficient or with the scrambled siRNA (control). The transfected worms were cultivated for up to 10 days and transfected again with siRNA1 on the fourth and seventh days; half of the medium was exchanged every day. After 10 days, all the eggs in the medium were collected and counted using light microscopy. The eggs in female uterus per couple were also counted using light microscopy. Adult male–female pairings were also observed and counted. The siRNA silencing effects and morphological changes were measured using qPCR and confocal laser scanning microscopy (TCS SP5; Leica, Mannheim, Germany), respectively.

**Table 2:** The sequences of siRNA (5′–3′)

	Sense	Anti-sense
*Sjfs800SiRNA1*	GCCCAUAGGAAMGGUAGAATT	UUCUACCAUUCCUAMGGGCTT
*Sjfs800SiRNA2*	GCCUCAUCUAUAUMGUCUATT	UAGACAAUAUAGAMGAGGCTT
*Sjfs800SiRNA3*	CCAAAGGUUAUCGMGGAAATT	UUUCCACGAUAACCUUMGGTT
*SiNCTRL*	UUCUCCGAACGMGUCACGUTT	ACGMGACACGUUCGGAGAATT
*SiCTRL*	ACAGUAGCAMGCAGCAGGTT	CCΜGCMGGCUACMGUTT

### Confocal laser scanning microscopy

The confocal laser scanning microscopy procedure has been described previously [22]. After 10 days of *Sjfs800-*specific siRNA1 treatment, the male–female paired couples were separated manually and fixed separately in a solution of 95% ethanol, 3% formaldehyde and 2% glacial acetic acid. The trematodes were then stained in 2.5% hydrochloric carmine (Ourchem, Shanghai, China) for 16 h at 37 °C and de-stained in 70% acidic ethanol. After dehydration in an ethanol series for 1 min in each concentration, parasites were cleared for 1 min each in 50% xylene diluted in ethanol and 100% xylene, and then whole-mounted with neutral balsam (Sinopharm Chemical, Shanghai, China) on glass slides. The reproductive organ morphology of the worms was examined using confocal laser scanning microscopy, with a 470-nm long pass filter and a 488-nm He/Ne laser under reflection mode. All images were obtained from at least three independent RNAi experiments using at least 7 female worms in each experiment.

### Statistical analysis

Results were acquired from three biological replicates representing three independent experiments with identical conditions. One-way analysis of variance and independent-sample t-tests were used for data analysis with the SPSS v.17.0, statistics software package. Data are expressed as the mean ± SEM, and *P* < 0.05 or *P* < 0.01 were deemed statistically significant.

## Results

### Number of paired and unpaired female worms on specific days after host infection

As well known, male–female pairing occurs up to 24 days after the host is infection. Before this, no male–female pairing occurring up to 16 days after the host is infected. Some pairing occurs 17 days post-infection (dpi), and pairing is common 18 dpi. The statistical methods and results are presented in Additional file 2: Figure S1 and Additional file 3: Table S2.

### Microarray screening of differentially expressed genes in female *S. japonicum* at pre-pairing (16 dpi), initial pairing (18 dpi) and oviposition (24 dpi)

The results of microarray analyses are shown in Additional file 4: Table S3, Additional file 5: Table S4 and Additional file 6: Table S5. To our knowledge the most comprehensive and informative probe assay design to date, indicated that after removing the duplicates, signal intensities were upregulated (FC ≥ 2) for nearly 132 sequences in female worms at 18 dpi and for nearly 198 sequences in female worms at 24 dpi. Many mRNA transcripts were differentially expressed at pre-pairing (16 dpi), initial pairing (18 dpi) and oviposition (24 dpi), with most of these genes elevated in expression oviposition. The most highly increased gene products at oviposition were associated primarily with oxygen metabolism, the metabolic machinery of egg production, and vitellarium development. The 30 differentially expressed genes with highest expression levels after pairing are given in Table 3.

**Table 3:** The top 30 differentially expressed genes in female *S. japonicum* in oviposition (24 dpi) relative to levels pre-pairing (16 dpi)

Probe name	Gene ID	Protein ID	*P*-value	Fold change	Description
CUST_14058_PI428956223	226474963	FN316039.1	0.001295	574.7126437	Extracellular superoxide dismutase [Cu-Zn]
CUST_13165_PI428956223	226473101	FN315505.1	0.021586	462.9629297	Trematode eggshell synthesis
CUST_21221_PI428956223	257207815	FN327835.1	0.020981	393.7007874	Protein matches (BLink) hsp40 subfamily A members 124
CUST_15773_PI428956223	226477693	FN316810.1	0.017062	252.525522	Unknown
CUST_21010_PI428956223	257207472	FN327620.1	0.029122	157.9778831	1 atypical protein kinase C
CUST_25908_PI428956223	257215731	FN330801.1	0.026122	149.7000599	DNA damage-responsive protein 48
CUST_12207_PI428956223	226471111	FN314905.1	0.04503	145.7725948	Serine/threonine kinase-1
CUST_4027_PI428956223	60600619	AY810878.1	0.038204	85.68980291	Female-specific protein 800
CUST_7243_PI428956223	56756748	AY814814.1	0.03629	71.73601148	Similar to histidine kinase DhkM
CUST_21060_PI428956223	257207522	FN327670.1	0.013621	44.98353526	Heterogeneous nuclear ribonucleoprotein
CUST_991_PI428956223	226469397	FN314445.1	0.018024	39.06715587	ESG-1 protein precursor
CUST_384_PI428962778	7644645	AW736781.	0.037435	36.43282441	Hemoglobin subunit alpha-1/2
CUST_15482_PI428956223	226477985	FN316955.1	0.035783	35.52397869	Prostatic spermine-binding protein precursor
CUST_11368_PI428956223	226469397	FN314445.1	0.026528	30.82413089	Annexin
CUST_5987_PI428956223	56753799	AY813365.1	0.039938	27.21671965	Transmembrane 9 superfamily member
CUST_15466_PI428956223	226477953	FN316939.1	0.000743	23.10313701	Unknown
CUST_7706_PI428956223	56757833	AY815325.1	0.02429	22.54660519	Putative epiplakin 1
CUST_7411_PI428956223	56757114	AY814997.1	0.025618	22.38696546	Contains repetitive sequences
CUST_999_PI428962772	56046805	CV738262.1	0.048425	22.00714633	Cytochrome *b*
CUST_713_PI428956223	60601043	AY811090.1	0.019922	21.02855408	Venom allergen-like (VAL) 7 protein
CUST_3699_PI428956223	60599676	AY810494.1	0.002295	17.84851112	Similar to labial-like protein
CUST_17613_PI428956223	226482651	FN318196.1	0.040935	8.663126238	PRP4 pre-mRNA processing factor 4 homolog B
CUST_17613_PI428956223	2290411	U92488.1	0.037768	8.470548849	Cyclophilin A
CUST_1037_PI428956223	22164072	AF412216.1	0.04818	8.237366919	Vacuolar proton translocating ATPase 116 kDa subunit a isoform 1

### Bioinformatics analysis of differentially expressed genes

In the biological process GO category, the genes involved in metabolic and biosynthetic processes were more active in initial pairing females (18 dpi) than pre-pairing (16 dpi), indicating that nutritional acquisition is more crucial for 18 dpi than 16 dpi female worms, which is likely a reflection of the oviposition status. In the molecular function GO category, the genes involved in hydrolase activity were more active in 18 dpi females and 24 dpi than 16 dpi females. In the cellular component GO category, gene products localized to membrane-bound organelles were more enriched in 24 dpi females than 16 dpi or 18 dpi females.

### Confirmation of differentially expressed genes by qPCR analysis

**Fig. 1:**
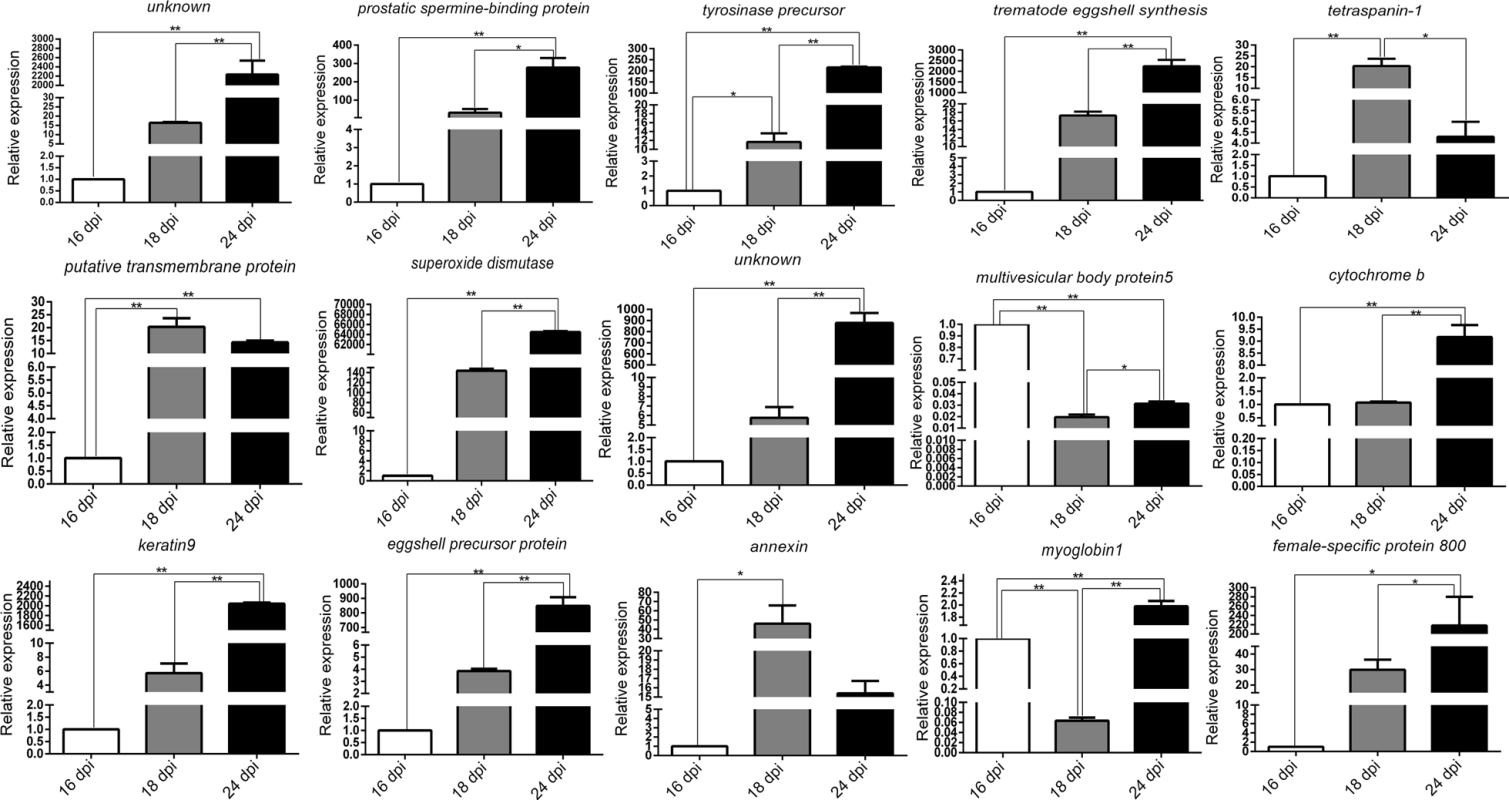
Validation of the microarray analysis results by qPCR. Data are normalized to the internal housekeeping control *PSMD4*. Expression levels of 15 selected genes were determined using the comparative method (2^−ΔΔct^). Data are expressed as the mean ± SEM of three independent experiments (three biological replicates). Asterisks show statistical differences (*P < 0.05, **P < 0.01) tested by one-way ANOVA with multiple comparisons (Tukey’s *post-hoc* test)

Fifteen differentially expressed genes were selected for confirmation using qPCR. The analysis for each gene was repeated three times and *PSMD4* was used as the housekeeping gene. After normalization, the relative changes in gene expression were determined using the 2^−ΔΔct^ method. The expression levels of two genes, *annexin* and *tetraspanin-1*, were not consistent between the qPCR and microarray analyses. The results for the remaining 13 gene expression levels were consistent across qPCR and microarray analyses (Fig. 1).

### Expression of *Sjfs800* in the eggs, cercariae and schistosomula at 16 dpi, worms at 24 and 42 dpi

**Fig. 2:**
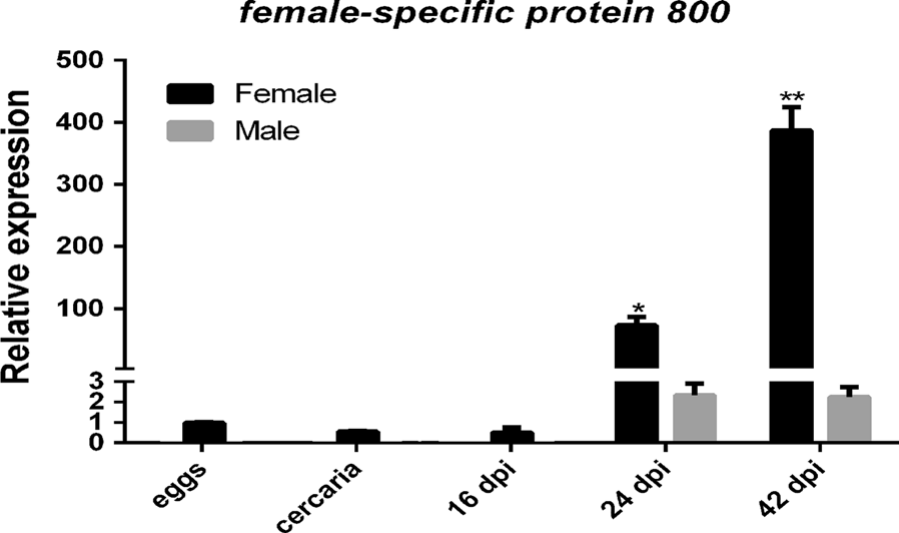
Expression levels of female-specific protein 800 (*fs800*) at various developmental stages of *S. japonicum* as measured by qPCR. qPCR was used to analyze *fs800* in life-cycle stages: eggs, cercariae, schistosomula (at 16 dpi) and adult worms at 24 and 42 dpi (*F*_*(4,10)*_ = 173.5, DF = 4, *P* < 0.0001). Data represent the mean ± SEM of three independent experiments (three biological replicates), compared to the egg group. *P < 0.05, **P < 0.01 by two-way ANOVA

The gene expression level for *Sjfs800* was determined using qPCR of the eggs, cercariae, schistosomula and adult worms at 24 and 42 dpi. We found *Sjfs800* that were highly expressed in females at 42 dpi. However, the expression levels of *Sjfs800* were very low in the eggs, cercariae and schistosomula at 16 dpi, and the male worms at 24 and 42 dpi. It modestly increased in female worms at 24 dpi, with a further increase at 42 dpi (Fig. 2). These results may be presumed to suggest that *Sjfs800* may be associated with sex maturation and oviposition of female worms.

### Localization by *in situ* hybridization of *Sjfs800* transcripts in *S. japonicum*

**Fig. 3:**
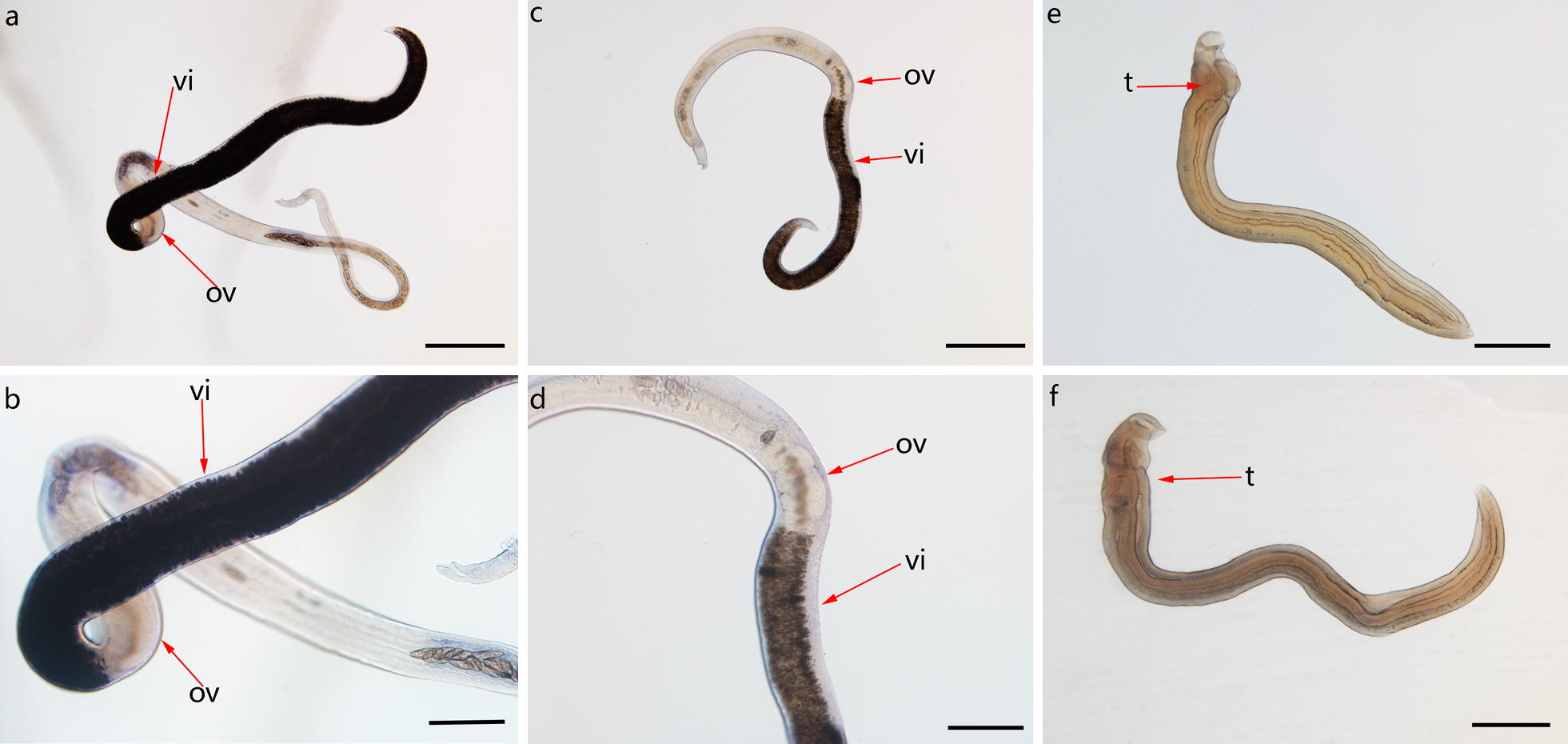
Localization of *Sjfs800* transcripts in *S. japonicum* at 24 dpi. Images are of whole-mount *in situ* hybridization results using DIG-labeled antisense (**a**, **b**, **e**) and sense (**c**, **d**, **f**) RNA probes of *Sjfs800*. *Sjfs800* transcripts in *S. japonicum* of 24 dpi males and females were detected using DIG-labeled RNA probes. Abundant transcription of *Sjfs800* was observed in the vitellarium. *Abbreviations*: ov, ovary; vt, vitellarium; t, testis. *Scale-bars*: **a**, **c**, **e**, 200 μm; **b**, **d**, **f**, 100 μm

An *in situ* hybridization analysis showed the transcriptional activities *Sjfs800* in *S. japonicum* at 24 dpi (Fig. 3). There were no positive signals in 24 dpi male worms. The expression of *Sjfs800* mRNA was observed only in the vitellarium in female worms.

### qPCR analysis of *Sjfs800* mRNA levels after *Sjfs800*-specific siRNA transfection in female *S. japonicum* at 28 dpi

**Fig. 4:**
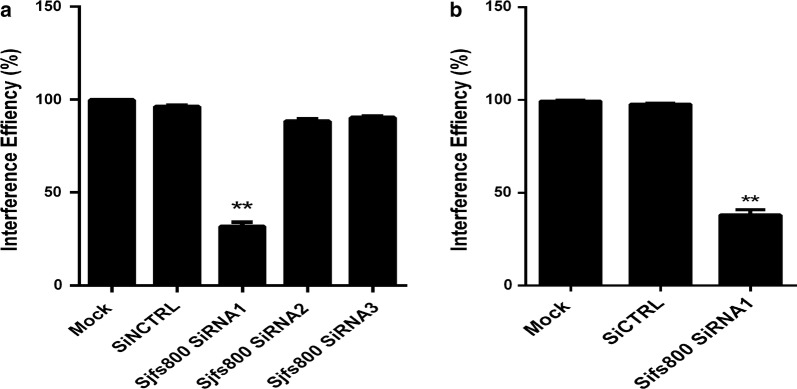
Effects of *Sjfs800*-specific siRNA transfection. **a** To determine the *Sfs800*-specific siRNA with the best interference efficiency *in vitro* as measured using qPCR, 28-dpi worms were transfected with one of three siRNAs (siRNA1-3) and were harvested 3 days later. The qPCR results showed the effects of siRNA1 were reduced by nearly 60% compared with those in the mock-transfected group (*F*_(4, 10)_ = 1525, *P* < 0.0001). **b** Effects of siRNA1 on *Sjfs800* mRNA levels in worms were tested 10 days later. *Sjfs800* mRNA levels were normalized to the endogenous control *SjPSMD4*. The qPCR analysis results showed that the *Sjfs800* mRNA levels in the group transfected with *Sjfs800* siRNA1 were reduced by approximately 60% compared with those in the scrambled siRNA-transfected group (*F*_(2, 6)_ = 1251, *P* < 0.0001). Asterisks show statistical differences (**P < 0.01) tested by one-way ANOVA, compared to the mock group. Data are expressed as the mean ± SEM of three independent experiments (three biological replicates)

The *Sjfs800* mRNA levels were analyzed by qPCR to determine the effects of the *Sjfs800*-specific siRNA transfection. First, the paired 28-dpi worms were transfected with one of the three siRNAs (siRNA1-3) targeting *Sjfs800*. After 3 days of cultivation, *Sjfs800* gene transcript levels were determined by qPCR. The reduction in *Sjfs800* transcription level following transfection with siRNA1 was 60% of that in the mock-transfected group and negative control group, which was the highest efficiency among the three siRNAs tested (Fig. 4a). Thus, *Sjfs800*-specific siRNA1 was used in the ensuing experiments. Worms were transfected with *Sjfs800*-specific siRNA1 or scrambled siRNA *in vitro*. The results of the qPCR analysis indicated that compared with the scrambled siRNA control group, the *Sjfs800*-specific siRNA1-treated group showed an approximately 60% reduction in *Sjfs800* mRNA levels on the 10 days later, and this experiment was repeated three times (Fig. 4b).

### Effects of *Sjfs800* knockdown on pairing rate, egg production and reproductive organ development

**Fig. 5:**
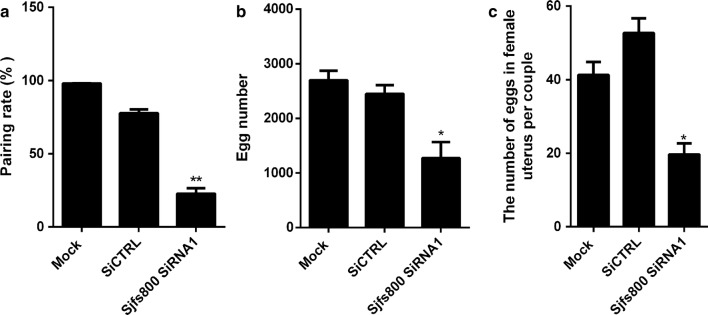
Effects of *Sjfs800* knockdown on pairing rate, egg production and reproductive organ development. **a** The male–female pairing rate of worms transfected with *Sjfs800* siRNA1 was reduced by approximately 70% compared with the rate in the scrambled siRNA-treated group (*F*_(2, 6)_ = 660.9, *P* < 0.0001). **b** The eggs in the culture medium were collected on day 10 and counted using light microscopy. SiNCTRL represents negative control siRNA group; SiCTRL represents scrambled siRNA (*F*_(2, 6)_ = 36.81, *P* = 0.0004). **c** The eggs in female uterus per couple were determined using light microscopy after 10 days of cultivation (*F*_(2, 6)_ = 66.59, *P* < 0.0001). Asterisks show statistical differences (*P < 0.05, **P < 0.01) tested by one-way ANOVA, compared to the mock group. Data are expressed as the mean ± SEM of three independent experiments three biological replicates

**Fig. 6:**
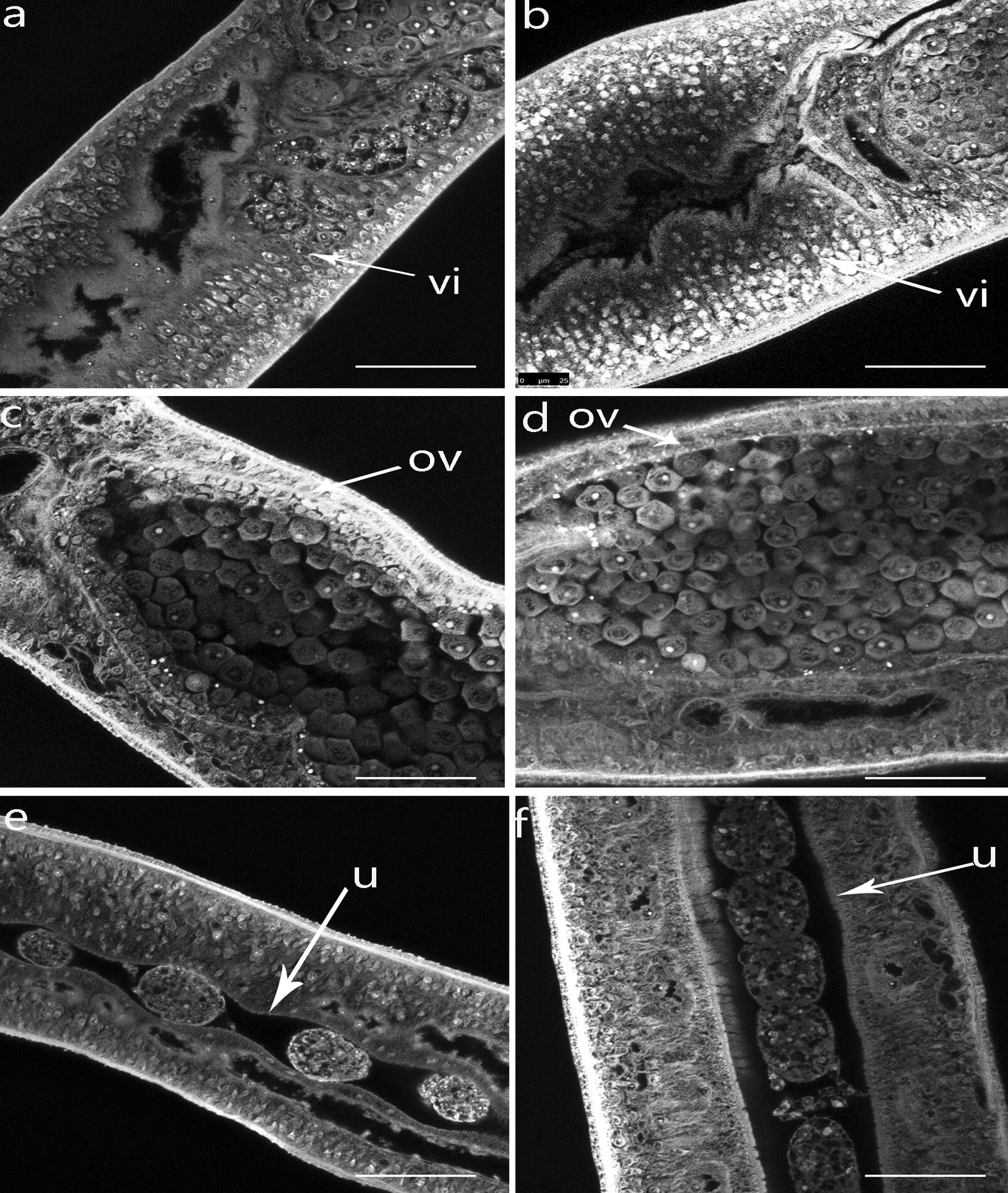
Confocal scanning laser microscopy images of the vitellarium in *S. japonicum* females after transfection with *Sjfs800* siRNA1. **a** Vitellarium from worms transfected with scrambled siRNA. **b** Vitellarium from worms transfected with *Sjfs800* siRNA1. Arrows in **a**, **b** point to vitelline follicles. **c** Ovary from worms transfected with scrambled siRNA. **d** Ovary from worms transfected with *Sjfs800* siRNA1. Arrow indicates ovary. **e** Uterus from worms transfected with scrambled siRNA; **f** Uterus from worms transfected with *Sjfs800* siRNA1. Arrows in **e**, **f** point to uterus. *Abbreviations*: vi, vitelline; ov, ovary; u, uterus. *Scale-bars*: 25 μm

The number of male–female paired worms was counted on the day 10 after siRNA transfection to determine the effect of knocking down the *Sjfs800* gene on the pairing rate. We found that the pairing rate in the *Sjfs800*-specific siRNA1-transfected group was significantly lower than that in the scrambled siRNA- transfected group (Fig. 5a). The numbers of paired worms in each group for each experiment are given in Additional file 7: Table S6. In addition, the number of eggs collected in the medium and counted using light microscopy was reduced approximately 50% in the *Sjfs800*-specific siRNA1 transfected group compared with that in the scrambled siRNA transfected group (Fig. 5b). After 10 days of *Sjfs800*-specific siRNA1 treatment, paired females were collected to count the number of eggs in the female uterus by using light microscopy. The results indicated that the number of eggs in female uterus per couple was also decreased approximately 50% compared with the group transfected with scrambled (Fig. 5c). The morphological changes in the vitellarium of *Sjfs800* siRNA1-treated worms were observed using confocal laser scanning microscopy. The vitellarium was well developed in female worms that were transfected with scrambled siRNA. By contrast, transfection with *Sjfs800* siRNA1 suppressed the development and maturation of the vitellarium, with fewer mature vitelline cells found in the *Sjfs800* siRNA1-treated females than in the controls (Fig. 6). The number of eggs was decreased in the uterus of female worms that were transfected with *Sjfs800* siRNA1 compared to the scrambled group. However, there were no significant ovarian morphology changes in the interference group compared to the scrambled group.

## Discussion

Paired adult female schistosomes produce a large number of eggs, which are primarily responsible for the schistosomiasis disease pathology and are critical for dissemination of the disease. The reproductive system of female schistosomes has been widely studied at the molecular level, and several studies have described transcriptomes that are differentially expressed before and after pairing in female schistosomes [16, 23–30]. For example, *SmFst* (follistatin of *S. mansoni*) was recently identified as a regulatory molecule in the transforming growth factor β pathway that is pairing dependently transcribed in the male gonad, likely facilitating processes leading to male competence [23]. Lu et al. [25] described pairing-induced processes within the gonads, including stem cell-associated and neural functions, by analyzing gonad-specific and pairing-dependent transcriptomes.

In the present study, gene microarray analysis was used to screen for differentially expressed genes pre-pairing and initial pairing females and females at oviposition of the *S. japonicum* female. We found the transcript profiles in 16-dpi females (pre-pairing) were similar to those at 18 dpi (initial pairing). However, substantial changes in gene expression were observed in 24-dpi females (oviposition), indicating that virgin female schistosomes undergo marked changes in gene expression before they complete maturation. Genes involved in reproduction, such as those associated with the cell cycle, egg formation and protein synthesis, were substantially upregulated in the adult female trematodes. The female gene expression patterns reflects that the female is ready to produce eggs after pairing which is consistent with findings of others [16, 23–30].

The vitellarium, which occupies the posterior two-thirds of the female schistosome body, produces vitellocytes. Vitellocytes supply nutrition to the developing zygote and constituents essential to egg shell construction. Mature vitellocytes join with fertilized oocytes in the ootype, which is where mature eggs are formed [31]. Thus, the development of the vitellarium plays essential roles in the production of schistosome eggs. Vitellarium is a flatworm-specific evolutionary innovation, so it could potentially be used as a specific therapeutics target [31].

Although the development of the vitellarium is a complicated process regulated by numerous molecules, an aim of the present study was to screen for some key molecules related to the development of the vitellarium. The results of our gene microarray analyses showed that the top 30 genes with respect to transcript abundance in 24-dpi females included representatives with proven roles in vitellarium development and egg production, including eggshell precursor protein, superoxide dismutase (*SOD*) and *Sjfs800*. In 2017, Wang et al. [16] mapped the dynamic transcriptome changes in male and female from pairing to maturation, to identify biogenic amines and insect-like hormones that regulate reproduction development in *S. japonicum*. They also found that eggshell precursor protein, *SOD* and *Sjfs800* were highly expressed in paired females. However, that study focused on biogenic amine neurotransmitters of males, which can control and maintain pairing with females by using the nervous system. In addition, insect-like hormones were shown to regulate the reproductive development of females [16]. In the present study, we aimed to determine the role of some of the genes related to vitellarium development in the production of schistosome eggs.

*Smfs800* gene was first found and identified by Reis et al. [32], who also used *in situ* hybridization to determine that *Smfs800* mRNA was expressed only in female vitelline cells, suggesting that *Sm*fs800 may play role in egg development. However, little is known about the functions of *Sjfs800* in vitellarium development and egg production. In the present study, S*jfs800* mRNA can be located in mature vitelline cells. Some developmental defects, especially a reduced number of mature vitelline cells in the vitellarium, were observed after *Sjfs800* gene knockdown by siRNA, and the pairing rate and oviposition rate were also significantly decreased. Therefore, we conclude that *Sj*fs800 is vital for vitelline cell development and maturation and that maturation of the vitellarium is required for *S. japonicum* females to produce eggs. The number male–female pairings was reduced by approximately 70% after *Sjfs800* siRNA transfection. The results of some studies have indicated that *Sj*fs800 may be a molecule downstream of *S. japonicum* Nanos1 or Abl tyrosine kinase activity [13, 33, 34]. However, the signaling pathway regulating vitellarium development centered on *fs800* still needs further study.

## Conclusions

In conclusion, our study showed substantial differences in the expression levels of some genes in the *S. japonicum* female before and after male–female pairing, including genes related to vitellarium development, which can affect pairing, sexual maturation and egg production. These results provide a deeper understanding of the reproductive biology of schistosomes and may lead to the development of novel approaches for the prevention and treatment of schistosomiasis.

## Supplementary information

**Additional file 1: Table S1.** Probe sequence selected for chip hybridization.

**Additional file 2: Figure S1.** Images of a 16 dpi female (left) and a 16 dpi male (right) under a light microscope.

**Additional file 3: Table S2.** Number of paired and unpaired female worms on specific days after host infection. In order to establish *Schistosoma japonicum* infection model, each Kunming mouse was infected with around 70 cercariae. After 14, 15 16, 17 and 18 days infection, mice were killed. The worms were perfused through the hepatic portal vein using standard perfusion techniques. Finally, the number of females that male–female pairing and male–female unpairing were counted separately.

**Additional file 4: Table S3.** 1138 sequences differentially expressed at initial pairing compared with the pre-pairing stage (FC ≥ 2).

**Additional file 5: Table S4.** 43,870 sequences differentially expressed during the oviposition stage compared with the pre-pairing stage (FC ≥ 2).

**Additional file 6: Table S5.** 6713 sequences differentially expressed during the oviposition stage compared with the initial pairing stage (FC ≥ 2).

**Additional file 7: Table S6.** The number of paired worms after RNA interference.

## Data Availability

The datasets supporting the results are included within the article and its additional files.

## Abbreviations

ANOVA: one-way analysis of variance
qPCR: real-time quantitative polymerase chain reaction
Ct: threshold cycle
PSMD4: 26S proteasome non-ATPase regulatory subunit 4
ISH: *in situ* hybridization
RNAi: RNA interference
CLSM: confocal laser scanning microscopy
