# Supplementary figures and images for: Differential gene expression, including Sjfs800, in Schistosoma japonicum females at pre-pairing, initial pairing and oviposition
Source: Parasit Vectors. 2019 Aug 23;12:414. doi: 10.1186/s13071-019-3672-8 (PMC6708146; doi:10.1186/s13071-019-3672-8)

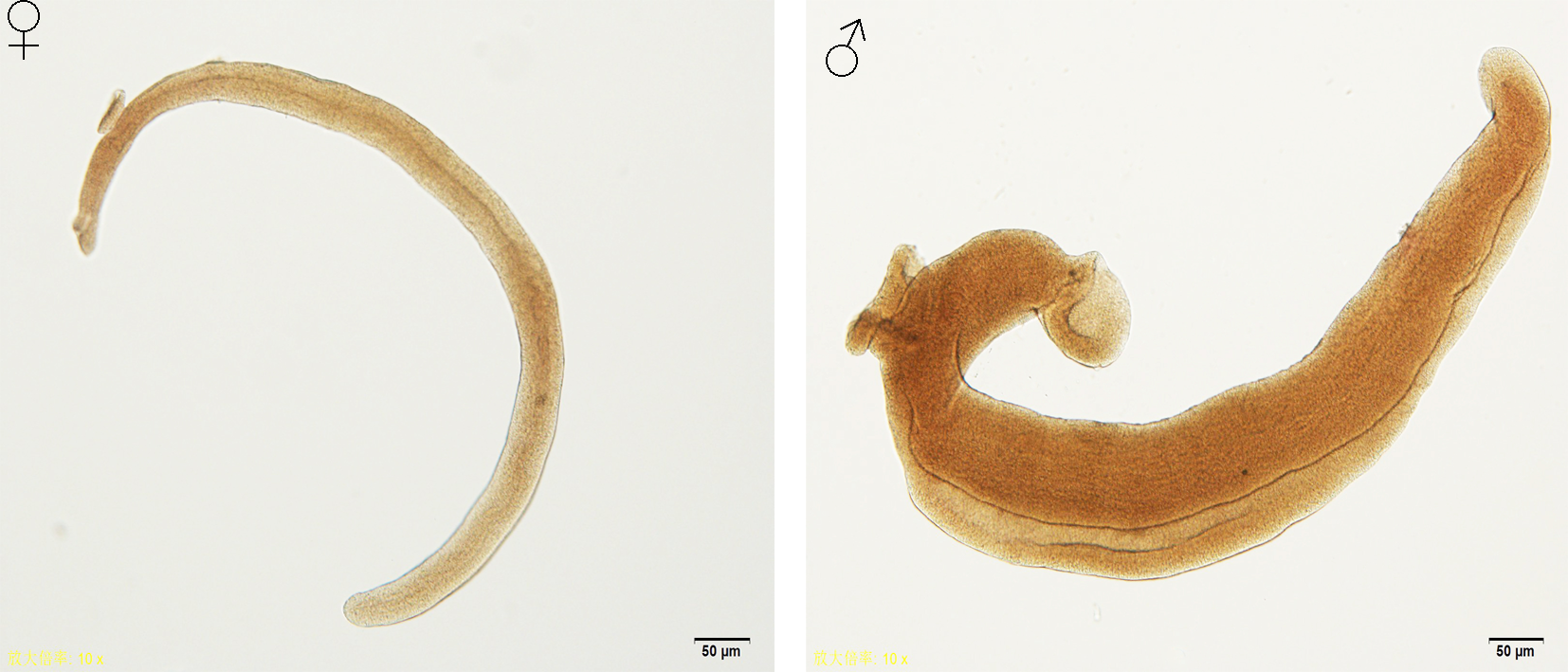

Supplement: Supplementary file 2 — Additional file 2: Figure S1. Images of a 16 dpi female (left) and a 16 dpi male (right) under a light microscope. [file 13071_2019_3672_MOESM2_ESM.tif]
