# Supplementary material for: Differential gene expression, including Sjfs800, in Schistosoma japonicum females at pre-pairing, initial pairing and oviposition
Source: Parasit Vectors. 2019 Aug 23;12:414. doi: 10.1186/s13071-019-3672-8 (PMC6708146; doi:10.1186/s13071-019-3672-8)
Supplement: Supplementary file 3 — Additional file 3: Table S2. Number of paired and unpaired female worms on specific days after host infection. In order to establish Schistosoma japonicum infection model, each Kunming mouse was infected with around 70 cercariae. After 14, 15 16, 17 and 18 days infection, mice were killed. The worms were perfused through the hepatic portal vein using standard perfusion techniques. Finally, the number of females that male–female pairing and male–female unpairing were counted separately. [file 13071_2019_3672_MOESM3_ESM.docx]

**Additional file 3：Table S2.** Number of paired and unpaired female worms on specific days after host infection. In order to establish *Schistosoma japonicum* infection model, each Kunming mouse was infected with around 70 cercariae. After 14, 15 16, 17 and 18 days infection, mice were killed. The worms were perfused through the hepatic portal vein using standard perfusion techniques. Finally, the number of females that male-female pairing and male-female unpairing were counted separately.

| Number of Female worms | Days Postinfection | | | |
| --- | --- | --- | --- | --- |
|  | 15 | 16 | 17 | 18 |
| Paired | 0±0 | 1±1 | 6±1 | 16±2 |
| Unpaired | 13±3 | 15±1 | 15±1 | 9±1 |
| Total | 13±3 | 16±1 | 21±2 | 25±3 |
