# Supplementary material for: Differential gene expression, including Sjfs800, in Schistosoma japonicum females at pre-pairing, initial pairing and oviposition
Source: Parasit Vectors. 2019 Aug 23;12:414. doi: 10.1186/s13071-019-3672-8 (PMC6708146; doi:10.1186/s13071-019-3672-8)
Supplement: Supplementary file 7 — Additional file 7: Table S6. The number of paired worms after RNA interference. [file 13071_2019_3672_MOESM7_ESM.docx]

| Groups | Pairing | Unpairing |
| --- | --- | --- |
| Mock | 12±2 | 3±2 |
| SiCTRL | 10±2 | 5±2 |
| Sifs800 siRNA1 | 3±1 | 12±1 |

**Additional file 7: Table S6.** The number of paired worms after RNA interference.
